# Determinants of uremic pruritus severity among patients undergoing chronic hemodialysis: A cross-sectional study

**DOI:** 10.1097/MD.0000000000049871

**Published:** 2026-07-24

**Authors:** Abdullah Hussien Alghamdi, Khalid Hamed Alhougail, Sulaiman Abdullah Alraqibah, Reem Hammad Albalawi, Laila Mohammed Mujarribi, Salman Alrasheed, Momen Hashim HajHassan Mohamed, Noor Abdulhakim Alfateel, Faisal Alzkari, Ziyad Alsulami, Musab Alsulami, Rama Abu Hassan, Renad Alsuhaibani, Abdullah Alaryni, Abdulrahman Mohammed Alanazi, Rayan A. Qutob, Abdullah Bukhari, Enad Alsolami, Abdulwahed A. Alotay, Meshal Alzakari

**Affiliations:** a Department of Internal Medicine, College of Medicine, Imam Mohammad Ibn Saud Islamic University (IMSIU), Riyadh, Saudi Arabia; b Department of Medicine, Dr. Sulaiman Al Habib Medical Group, Riyadh, Saudi Arabia; c College of Medicine, Imam Mohammad Ibn Saud Islamic University (IMSIU), Riyadh, Saudi Arabia; d Department of Medicine, College of Medicine, Imam Mohammad Ibn Saud Islamic University (IMSIU), Riyadh, Saudi Arabia; e Department of Internal Medicine, College of Medicine, University of Jeddah, Jeddah, Saudi Arabia.

**Keywords:** chronic kidney disease, hemodialysis, itching, Saudi Arabia

## Abstract

Uremic pruritus, also called chronic kidney disease-associated Pruritus (CKD-aP), is a symptom of severe and ongoing itching that patients with CKD or end-stage renal disease frequently experience. The purpose of this study was to evaluate the factors associated with moderate-to-severe uremic pruritus in patients receiving chronic hemodialysis. This cross-sectional study was conducted at Dr Sulaiman Al-Habib Hospital in Riyadh, Saudi Arabia, from June to October 2025. The severity of itching was assessed using a single-item scale from 0 to 10, with 0 indicating no itching and 10 indicating maximum itching. Responses of >0 were summed to obtain itching intensity. Among participants, 53 (57.0%) were male. The prevalence of severe itching among participants was 10.8%, with a mean itching scale score of 2.35 ± 2.85 out of 10. The noncitizen participants showed higher itching scores than Saudi patients, with a difference that reached the threshold of statistical significance (4.00 ± 3.43) and (2.18 ± 2.75), respectively. Multivariable logistic regression identified that females were 4 times more likely to have moderate-to-severe itching than males (aOR: 5.64 [95% CI: 1.39–22.99]). The majority of hemodialysis patients had minor uremic pruritus. Further research involving larger samples is also warranted to investigate the factors contributing to uremic pruritus in hemodialysis patients.

## 1. Introduction

Uremic pruritus, also called Chronic Kidney Disease-associated Pruritus (CKD-aP), is a symptom of severe and ongoing itching that patients with CKD or end-stage renal disease frequently experience.^[1]^ The desire to scratch in the absence of skin lesions often occurs daily, either as a localized itch that affects the arms, face, and back, or as a generalized itch.^[2]^ It can be experienced at any stage of CKD; however, Stage 5 CKD carries a 19% higher risk of having it than Stage 3.^[3]^ Additionally, this pruritus is more common in hemodialysis patients (37–70%) and peritoneal dialysis patients (29–71%) than in non-dialytic CKD patients (19–34%).^[3-6]^ Uremic pruritus can significantly impair patients’ sleep, quality of life, and medication adherence, and trigger depressive symptoms,^[7,8]^ highlighting the need to investigate factors associated with uremic pruritus and subsequently implement tailored strategies to reduce its burden.

Many factors may contribute to the development of pruritus in CKD, including abnormalities in calcium and phosphorus metabolism.^[9]^ Pruritus in hemodialysis patients has been linked to elevated serum calcium and phosphorus levels.^[10]^ Experimental results from mice showed that calcium phosphate injections induced pruritus by producing IL-6.^[11]^ Furthermore, high-sensitivity C-reactive protein and pruritus were found to be significantly correlated in hemodialysis patients.^[12]^ A multicentric study of hemodialysis patients found that more severe uremic pruritus was associated with longer hemodialysis duration, absence of vitamin D supplementation, lower albumin levels, and high-sensitivity C-reactive protein levels. Furthermore, older hemodialysis patients and those with dry skin were 3.9 and 9.9 times more likely to experience extremely severe pruritus, respectively.^[13]^ Male patients receiving peritoneal dialysis experienced more severe uremic pruritus during the day, whereas topical drug use and the length of peritoneal dialysis were associated with more severe pruritus at night.^[14]^

The inconsistent findings regarding the factors associated with uremic pruritus underscore the need for additional research to identify the true associated variables despite the variations in study design, populations and settings. Thus, the purpose of this study was to evaluate factors associated with moderate-to-severe uremic pruritus in patients receiving chronic hemodialysis. The results of this study will contribute to the body of knowledge on factors affecting uremic pruritus and guide future CKD management strategies to reduce its incidence and improve CKD patients’ clinical outcomes.

## 2. Materials and methods

### 2.1. Study design

This cross-sectional study was conducted at Dr Sulaiman Al-Habib Hospital in Riyadh, Saudi Arabia, from June to October 2025.

### 2.2. Study population

This research was conducted in the hemodialysis unit at Dr Sulaiman Al-Habib Hospital in Riyadh, a fully equipped tertiary care facility serving a diverse patient demographic with chronic renal disease. The study population comprised adult patients aged 18 years and older receiving hemodialysis. Patients were invited to participate in this study during their routine dialysis sessions over the study period.

The inclusion criteria were patients aged 18 years and older who were diagnosed with CKD-associated pruritus by a dermatologist. Exclusion criteria included patients with a primary dermatological disease such as eczema, those with known allergic conditions or drug-induced pruritus, or those with hematologic disorder-associatedpruritus. Furthermore, patients who refused to consent or were unable to communicate were excluded from this study.

### 2.3. Instrument

Data were collected using the validated Worst Itch Numeric Rating Scale (WI-NRS) Questionnaire CKD-AP to assess the severity of pruritus associated with chronic kidney disease. We approached all eligible patients on hemodialysis at Dr Sulaiman Al-Habib Hospital in Riyadh during regular dialysis sessions. The participants were asked to grade the severity of their pruritus, based on the worst itch intensity they had experienced in the past 24 hours, with scores ranging from 0 to 10, where 0 means no itch and 10 reflects the worst itch imaginable. A trained researcher or nurse provided the questionnaire to ensure that patients were properly instructed and answered correctly. Severity of itching categories were based on the cutoffs of the WI-NRS scale, where a score of 1 to 3 indicated mild pruritus, 4 to 6 indicated moderate pruritus, and 7 to 10 indicated severe pruritus.

### 2.4. Data analysis

Data were analyzed using the Statistical Package for the Social Sciences (SPSS) version 27 (IBM). Descriptive statistics were presented as mean and standard deviation for continuous variables, and frequency and percentage for categorical variables. Normality of continuous variables, such as the itching score, was assessed using the Shapiro–Wilk test. An independent *t*-test and analysis of variance were applied since the data met the normality assumptions. Multiple variable logistic regression analysis was used to identify predictors of moderate-to-severe itching. The first regression model used the WI-NRS cutoff point for moderate-to-severe itching. In the second regression model, the mean itching score (2.35; standard deviation: 2.85) was used to define the dummy variable. A *P*-value < .05 was considered statistically significant for the whole analysis. This research did not have missing data for the patients.

### 2.5. Ethical approval

This study was approved by the Research Center at Dr Suliman Al-Habib Hospital, Riyadh, Saudi Arabia (RC25.11.109).

### 2.6. Sample size calculation

Due to the exploratory nature of this research, no formal a priori sample size was estimated. All patients who met the inclusion criteria and consented to participate were involved in this research.

## 3. Results

Among participants, 53 (57.0%) were male and 40 (43.0%) were female. The majority were Saudi nationals (84, 90.3%) resident in the central region (66, 71.0%). Educational levels varied, with 33 (35.5%) having less than a high school education and 16 (17.2%) having graduated from high school. Employment status included 25 (26.9%) employed and 37 (39.8%) unemployed participants; see Table 1.

**Table 1:** The sociodemographic characteristics of participants.

Variable		N	%
Gender	Male	53	57.0
	Female	40	43.0
Nationality	Non-KSA	9	9.7
	KSA	84	90.3
Region of residence	Central	66	71.0
	Western	11	11.8
	Eastern	3	3.2
	Northern	2	2.2
	Southern	11	11.8
Educational level	Less than high school	33	35.5
	High school graduate	16	17.2
	Diploma	17	18.3
	Bachelor’s degree	20	21.5
	Postgraduate degree	7	7.5
Employment status	Employed	25	26.9
	Unemployed	37	39.8
	Student	2	2.2
	Retired	29	31.2

KSA = Kingdom of Saudi Arabia.

The majority of patients had undergone dialysis for 1 to 5 years (46, 49.5%), followed by less than 1 year (26, 28.0%). In terms of dialysis access, 40 (43.0%) participants utilized an arteriovenous fistula (AVF). Only 1 patient employed an arteriovenous graft (AVG) (1.1%), and 52 individuals (55.9%) used a catheter for dialysis access. Calcium levels were normal in 47 patients (50.5%), whereas parathyroid hormone (PTH) levels ranged from 14 to 63 pg/mL in 50 patients (53.8%). Over half of the participants (54, 58.1%) used sevelamer, 57 (61.3%) used calcium, 29 (31.2%) used cinacalcet, and 55 (59.1%) used vitamin D. The prevalence of severe itching among participants was 10.8%, with a mean itching scale score of 2.35 ± 2.85 out of 10; see Table 2.

**Table 2:** Clinical characteristics and dialysis-related factors among study participants.

Variable		N	%
Years on dialysis	<1	26	28.0
	1–5	46	49.5
	5–10	17	18.3
	>10	4	4.3
Access of dialysis	AVF	40	43.0
	AVG	1	1.1
	Catheter	52	55.9
Number of HD sessions missed per month	1–2	85	91.4
	3–4	8	8.6
	5–6	0	0.0
	>=7	0	0.0
Hb level	<=8	8	8.6
	8–10	28	30.1
	10–12	49	52.7
	>12	8	8.6
PO4	Low	7	7.5
	Normal	47	50.5
	High	39	41.9
Ca	Low	44	47.3
	Normal	47	50.5
	High	2	2.2
PTH	114	11	11.8
	14–63	50	53.8
	Above 63	32	34.4
Kt/v	<1.2	17	18.3
	1.2–1.4	45	48.4
	>1.4	31	33.3
Using of sevelamer	No	39	41.9
	Yes	54	58.1
Using of calcium	No	36	38.7
	Yes	57	61.3
Using of cinacalcet	No	64	68.8
	Yes	29	31.2
Using any type of Vitamin D	No	38	40.9
	Yes	55	59.1
Based on your experience, which group describes your itch severity best?	Mild	71	76.3
	Moderate	12	12.9
	Severe	10	10.8

AVF= arteriovenous fistula, AVG = arteriovenous graft, Hb = hemoglobin, HD = hemodialysis, Kt/v = dialysis adequacy index, PO4 = phosphate, PTH = parathyroid hormone.

The mean itching score of 2.35 ± 2.85 demonstrated mid-intensity itching among participants. The noncitizen participants showed higher itching scores than Saudi patients, with a difference that reached the threshold of statistical significance (4.00 ± 3.43) and (2.18 ± 2.75), respectively; see Table 3.

**Table 3:** The mean itching intensity score stratified by patients’ sociodemographic characteristics.

Variable		Itching score	
		Mean ± SD	*P*-value
Gender	Male	1.92 ± 2.72	.09
	Female	2.93 ± 2.96	
Nationality	Non-KSA	4.00 ± 3.43	.05
	KSA	2.18 ± 2.75	
Region of residence	Central	2.41 ± 2.82	.16
	Western	1.18 ± 1.78	
	Eastern	0.00 ± 0.00	
	Northern	4.00 ± 5.66	
	Southern	3.55 ± 3.42	
Educational level	Less than high school	2.33 ± 2.71	.29
	High school graduate	1.75 ± 2.65	
	Diploma	2.65 ± 3.30	
	Bachelor’s degree	3.20 ± 3.17	
	Postgraduate degree	0.71 ± 0.76	
Employment status	Employed	2.84 ± 3.50	.25
	Unemployed	2.73 ± 2.69	
	Student	1.00 ± 1.41	
	Retired	1.55 ± 2.38	
Years on dialysis	<1	2.04 ± 2.78	.88
	1–5	2.43 ± 3.14	
	5–10	2.71 ± 2.52	
	>10	2.00 ± 1.41	
Access of dialysis	AVF	3.00 ± 3.05	.16
	AVG	2.00 ± 0.00	
	Catheter	1.87 ± 2.65	
Number of HD sessions missed per month	1–2	2.38 ± 2.82	.81
	3–4	2.13 ± 3.44	

AVF= arteriovenous fistula, AVG = arteriovenous graft, HD = hemodialysis, KSA = Kingdom of Saudi Arabia, SD = standard deviation.

Multiple variable logistic regression analysis was used to identify predictors of moderate-to-severe itching intensity among the participating patients. Using the WI-NRS Questionnaire cutoff point, the regression model identified that females were 5 times more likely to have severe itching than males (aOR: 5.64 [95% CI: 1.39–22.99]), *P*-value = 0.016, Table 4. To verify the robustness of the study findings, another multiple logistic regression analysis that used the mean itching score of 2.35 ± 2.85 of the study sample as the cutoff point for the regression analysis identified that females were 4 times more likely to have severe itching than males (aOR: 4.08 [95% CI: 1.26–13.18]); see Table 5.

**Table 4:** Predictors of moderate-to-severe itching using WI-NRS Questionnaire cutoff point.

Variables	B-coefficient	Significance level	Adjusted odds ratio	95% CI		
				Lower limit	Upper limit	
Gender						
Male	1.00					
Female	1.73	**0.016**	5.64	1.39		22.99
Nationality						
Non-KSA	1.00					
KSA	-0.280	0.759	0.76	0.13		4.51
Region of residence						
Central	1.00					
Noncentral	−0.366	0.530	0.69	0.22		2.17
Educational level						
Less diploma	1.00					
Diploma or higher	0.973	0.20	2.65	0.60		11.67
Employment status						
Employed	1.00					
Others	−0.073	0.92	0.93	0.22		3.90

Bold indicates *P* < .05.

CI = confidence interval, KSA = Kingdom of Saudi Arabia.

**Table 5:** Predictors of itching intensity.

Variables	B-coefficient	Significance level	Adjusted odds ratio	95% CI		
				Lower limit	Upper limit	
Gender						
Male	1.00					
Female	1.405	**0.019**	4.08	1.26		13.18
Nationality						
Non-KSA	1.00					
KSA	0.254	0.765	1.29	0.25		6.79
Region of residence						
Central	1.00					
Noncentral	−0.455	0.378	0.63	0.23		1.75
Educational level						
Less diploma	1.00					
Diploma or higher	0.502	0.430	1.65	0.47		5.75
Employment status						
Employed	1.00					
Others	−0.301	0.63	0.74	0.22		2.55

CI = confidence interval, KSA = Kingdom of Saudi Arabia.

## 4. Discussion

The purpose of this study was to evaluate uremic pruritus and its associated factors among patients receiving chronic hemodialysis. Overall, 10.8% of patients suffered from severe itching. Compared with Saudi patients, noncitizens had higher itching scores, with a difference that reached the threshold of statistical significance. Furthermore, multivariable logistic regression analysis identified that females were 4 times more likely to have severe itching than males.

Nearly half of the participants in the present study had been on dialysis for 1–5 years (49.5%). This finding aligns with that of a Chinese study, where 50.7% of hemodialysis patients had a dialysis vintage of 1–5 years.^[15]^ Studies carried out in Saudi Arabia and Iran (59.3%) revealed a greater percentage of hemodialysis patients with the same dialysis period (63.9–80.4%), whereas a lower proportion was observed in an Egyptian study (38.6%).^[16-19]^ Therefore, to improve clinical outcomes and reduce complications among hemodialysis patients, customized treatment plans must evaluate the distribution of dialysis vintage and the factors that influence it.

Only 43.0% and 1.1% of patients in the current study utilized AVF and AVG, respectively, whereas the majority (55.9%) used a catheter for dialysis access. High catheter use for hemodialysis was also observed in earlier studies.^[20-22]^ In accordance with clinical practice guidelines, AVF is the preferred vascular access method for hemodialysis patients. AVG is typically used in cases where a surgeon is unable to place an AVF, while catheters are the least preferred because their use has been linked to an increased risk of myocardial infarction, stroke, peripheral vascular disease, and mortality.^[23,24]^ Moreover, a comprehensive review found that the use of catheters for hemodialysis was associated with the highest risk of death, infections, and cardiovascular events when compared to alternative vascular access options, whereas fistulas were linked to the lowest risk.^[25]^ Future programs should therefore concentrate on early referral for the creation of dialysis access, emphasize the benefits of AVF, and enhance staff and patient education to lessen dependency on catheters and the complications associated with them.^[26]^

Nearly half of the participants in the current study had low serum calcium levels (47.3%), and about a third had PTH levels above the normal range (>63 pg/mL, 34.4%). These findings may point to secondary hyperparathyroidism, the excessive PTH secretion caused by parathyroid gland hyperplasia, usually brought on by hypocalcemia, hyperphosphatemia, or low levels of active vitamin D.^[27]^ In a prior Argentine study among CKD-dialysis patients, only half of the participants had normal calcium levels (51.6%), but substantially higher PTH levels than in our study, with 54.5% and 28.3% having iPTH levels > 300 pg/mL and >600 pg/mL, respectively.^[28]^ Other studies have also reported that the majority of dialysis patients had PTH levels exceeding 150 pg/mL.^[29,30]^ The relatively lower prevalence of high PTH observed in our study may relate to improved monitoring and management strategies for maintaining mineral balance in CKD patients. Regarding medication use, more than half of patients in our study were receiving sevelamer (58.1%), calcium supplements (61.3%), and vitamin D (59.1%) to manage bone-mineral disturbances associated with CKD. Approximately a third were using cinacalcet (31.2%), which is typically reserved for patients with persistent hyperparathyroidism despite standard therapy.^[31]^ Despite these findings, a significant percentage of patients were not receiving the recommended medications, emphasizing the need for improved patient education, individualized treatment regimens, and better adherence to CKD management guidelines.

According to the current study, 10.8% of the patients reported severe itching, and the average itching severity score was 2.35 ± 2.85 (on a 0–10 scale), indicating mild symptom intensity. A prior Turkish study of hemodialysis patients revealed a similar prevalence of pruritus (49.3%).^[32]^ Nonetheless, with a mean score of 13.97 ± 4.11, the patients reported moderate severity. A large Italian survey found that 64% of hemodialysis patients who provided their itch score suffered from moderate-to-severe itching.^[33]^ In Canada, among patients who reported having pruritus (57%), 31% were classified as having moderate-to-severe pruritus.^[34]^ In the United States, a study by Thompson et al identified that around 49.0% of patients with CKD reported CKD-associated uremic pruritus of moderate itching severity, and 30.0.% of the patients reported severe CKD-associated uremic pruritus.^[35]^ According to a recent review, the prevalence of uremic pruritus among patients on dialysis varied from 28% to 70%.^[9]^ Diverse patient characteristics and risk factors may explain this, including variations in the diagnostic criteria and evaluation instruments employed, potential medication nonadherence, and patient underreporting of symptoms.^[36,37]^ Factors such as patient-related factors, variations in the type and duration of dialysis, and mineral balance control could also contribute to variation in itching severity, with worsening symptoms, lower quality of life, and poorer sleep quality happening with more severe itching.^[37]^ A previous study by Helou et al in 2025 examined factors associated with CKD-associated pruritus and identified that higher phosphorus levels, depression, smoking, and polypharmacy were factors associated with increased CKD-associated pruritus severity.^[38]^ Even minor pruritus can negatively affect mood, sleep, and quality of life, underscoring the importance of consistent symptom monitoring to optimize patient care.^[39,40]^ Moreover, CKD-associated pruritus is associated with increased dialysis treatment time, an increased possibility of withdrawing from dialysis, and missing dialysis sessions.^[5,41]^

The current study found that noncitizen participants had higher itching scores than Saudi patients, with a marginally significant difference. One potential hypothesis that could explain this finding is socioeconomic status differences and the extent of access to healthcare resources. Due to limited insurance coverage, limited access to specialized nephrology treatment, or financial constraints, noncitizens may not receive the best care for CKD-related disorders, including uremic pruritus.^[42]^ However, our study did not examine the role of these variables; therefore, this finding should be interpreted carefully. Moreover, further research in larger samples is essential to confirm this association, as the sample size for this subgroup was very small.

This study found that females were 5 times more likely to have severe itching than males. A previous study by Ständer et al identified gender-specific differences in the location, quality, and triggering of chronic pruritus, in the underlying disease, and in scratching behavior.^[43]^ Furthermore, Ständer et al reported that females more often showed chronic scratch lesions and prurigo nodularis and had more neuropathic and psychosomatic diseases underlying their pruritus.^[43]^ A previous study by Rehman et al did not identify a statistically significant association between patients’ gender and CKD-AP.^[44]^ On the other hand, other research identified that female gender is associated with a higher risk of CKD-AP.^[41,45]^

This study has limitations. The cross-sectional study design limits the ability to examine causality among the study variables. Furthermore, the itching score is a bounded 0 to 10 variable, and several subgroup comparisons appear unstable because of very small group sizes, including nationality, vascular access, and residence categories. This research did not examine the number of patients assessed, excluded, or refusing participation, nor the timing of pruritus assessment relative to dialysis sessions and laboratory testing, or confounders such as xerosis, liver disease, dialysis adequacy, inflammatory status, comorbidity burden, dialysis vintage, access type, mineral metabolism, or medication-related factors, and these were not fully addressed in the adjusted analysis. Furthermore, this research included only patients with CKD-AP. Therefore, the prevalence rate of CKD-AP among patients on hemodialysis could not be estimated. Therefore, the study findings should be interpreted carefully.

## 5. Conclusion

The majority of hemodialysis patients had mid-itching intensity. Although this correlation was only marginally significant, noncitizen patients had higher itching scores than Saudi patients. Similarly, females showed higher odds of having moderate-to-severe itching compared to males. Given the primarily sociodemographic adjustment set, these findings should be evaluated cautiously. Patient education, enhancing medication adherence, routine symptom assessment, and tailored management techniques are crucial to reduce the intensity of uremic pruritus and, consequently, enhance patient outcomes and quality of life in this population. Further research involving larger samples and clinically important covariates is also warranted to investigate the factors associated with uremic pruritus in hemodialysis patients.

## Author contributions

**Conceptualization:** Abdullah Hussien Alghamdi.

**Data curation:** Abdullah Hussien Alghamdi.

**Formal analysis:** Abdullah Hussien Alghamdi.

**Funding acquisition:** Abdullah Hussien Alghamdi.

**Investigation:** Abdullah Hussien Alghamdi, Khalid Hamed Alhougail, Sulaiman Abdullah Alraqibah, Reem Hammad Albalawi, Laila Mohammed Mujarribi, Salman Alrasheed, Momen Hashim HajHassan Mohamed, Noor Abdulhakim Alfateel, Faisal Alzkari, Ziyad Alsulami, Musab Alsulami, Rama Abu Hassan, Renad Alsuhaibani, Abdullah Alaryni, Abdulrahman Mohammed Alanazi, Rayan A. Qutob, Abdullah Bukhari, Enad Alsolami, Abdulwahed A. Alotay, Meshal Alzakari.

**Methodology:** Abdullah Hussien Alghamdi.

**Project administration:** Abdullah Hussien Alghamdi.

**Resources:** Abdullah Hussien Alghamdi, Khalid Hamed Alhougail, Sulaiman Abdullah Alraqibah, Reem Hammad Albalawi, Laila Mohammed Mujarribi, Salman Alrasheed, Momen Hashim HajHassan Mohamed, Noor Abdulhakim Alfateel, Faisal Alzkari, Ziyad Alsulami, Musab Alsulami, Rama Abu Hassan, Renad Alsuhaibani, Abdullah Alaryni, Abdulrahman Mohammed Alanazi, Rayan A. Qutob, Abdullah Bukhari, Enad Alsolami, Abdulwahed A. Alotay, Meshal Alzakari.

**Software:** Abdullah Hussien Alghamdi.

**Supervision:** Abdullah Hussien Alghamdi.

**Validation:** Abdullah Hussien Alghamdi.

**Visualization:** Abdullah Hussien Alghamdi.

**Writing – original draft:** Abdullah Hussien Alghamdi.

**Writing – review & editing:** Abdullah Hussien Alghamdi, Khalid Hamed Alhougail, Sulaiman Abdullah Alraqibah, Reem Hammad Albalawi, Laila Mohammed Mujarribi, Salman Alrasheed, Momen Hashim HajHassan Mohamed, Noor Abdulhakim Alfateel, Faisal Alzkari, Ziyad Alsulami, Musab Alsulami, Rama Abu Hassan, Renad Alsuhaibani, Abdullah Alaryni, Abdulrahman Mohammed Alanazi, Rayan A. Qutob, Abdullah Bukhari, Enad Alsolami, Abdulwahed A. Alotay, Meshal Alzakari.
